# Anisotropic silicon nanowire arrays fabricated by colloidal lithography

**DOI:** 10.1039/d1na00259g

**Published:** 2021-05-10

**Authors:** Marcel Rey, Fedja Jan Wendisch, Eric Sidney Aaron Goerlitzer, Jo Sing Julia Tang, Romina Sigrid Bader, Gilles Remi Bourret, Nicolas Vogel

**Affiliations:** Institute of Particle Technology, Friedrich-Alexander University Erlangen-Nürnberg Cauerstrasse 4 91058 Erlangen Germany Nicolas.Vogel@fau.de; Department of Chemistry and Physics of Materials, University of Salzburg Jakob Haringer Strasse 2A A-5020 Salzburg Austria Gilles.Bourret@sbg.ac.at; Department of Physics and Astronomy, The University of Edinburgh Mayfield Road Edinburgh EH9 3JZ UK; Department of Biofunctionalized Materials and (Glyco)Biotechnology, Fraunhofer Institute for Applied Polymer Research IAP Geiselbergstr. 69 14476 Potsdam Germany; Chair of Polymer Materials and Polymer Technologies, Institute of Chemistry, University of Potsdam 14476 Potsdam-Golm Germany

## Abstract

The combination of metal-assisted chemical etching (MACE) and colloidal lithography allows for the affordable, large-scale and high-throughput synthesis of silicon nanowire (SiNW) arrays. However, many geometric parameters of these arrays are coupled and cannot be addressed individually using colloidal lithography. Despite recent advancements towards higher flexibility, SiNWs fabricated *via* colloidal lithography and MACE usually have circular, isotropic cross-sections inherited from the spherical templates. Here we report a facile technique to synthesize anisotropic SiNWs with tunable cross-sections *via* colloidal lithography and MACE. Metal films with an elliptical nanohole array can form from shadows of colloidal particles during thermal evaporation of the metal at tilted angles. The aspect ratio of these anisotropic holes can be conveniently controlled *via* the deposition angle. Consecutive MACE using these patterned substrates with or without prior removal of the templating spheres results in arrays of anisotropic SiNWs with either elliptical or crescent-shaped cross-sections, respectively. As a consequence of the anisotropy, long SiNWs with elliptical cross-sections preferentially collapse along their short axis, leading to a controlled bundling process and the creation of anisotropic surface topographies. These results demonstrate that a rich library of SiNW shapes and mesostructures can be prepared using simple spherical colloidal particles as masks.

## Abstract 2

We demonstrate the design of silicon nanowire arrays with tunable elliptical cross-sections. Their anisotropic character of the individual pillars affords defined bending or bundling of the arrays.

## Graphical abstract

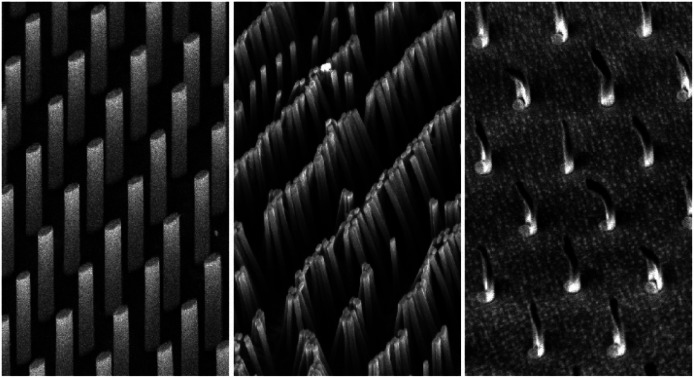

## Introduction

Nanoscale surface patterning is used in various scientific disciplines to provide structured materials with new functional properties. An example of such nanostructures are vertically-aligned silicon nanowire (SiNW) arrays, which have outstanding and tunable optical properties such as light trapping,^1,2^ waveguiding,^3^ Mie resonances,^4^ and diffractive effects.^5^ These tunable properties, in turn, are interesting for various applications such as photovoltaics,^2,6,7^ photocatalysis,^8,9^ and sensing.^3–5,10^ Additionally, SiNW arrays can serve as nano-bio cellular interfaces, which have recently emerged as a powerful tool for cellular manipulations and interrogations^11,12^ as well as gene delivery.^13–17^ The properties of SiNW arrays depend on their crystallographic structure (*e.g.* polycrystalline, single-crystalline) as well as on their geometry (shape, diameter, height and spacing).^3–5,16,18–20^ Thus, ideal SiNW fabrication methods should not only be robust, scalable and affordable, but should also provide exact control over their geometrical parameters.

SiNWs can be synthesized *via* bottom-up approaches, such as vapor–liquid–solid (VLS) synthesis^21–23^ or *via* top-down processes, such as reactive ion etching (RIE)^24^ or metal-assisted chemical etching (MACE).^25,26^ The synthesis of nanowire arrays with controlled geometry in the sub-micron realm requires the use of high resolution lithographical techniques such as deep-UV photolithography,^27^ electron-beam lithography^28^ or dip-pen nanolithography.^29^ These techniques are able to produce nanostructured masks with flexible geometrical motives. However, these fabrication processes are typically sequential and thus limited to small areas, or require expensive equipment and clean-room facilities.

Colloidal lithography has emerged as a simple alternative to prepare large-area 2D nanoscale patterns^30,31^ and its combination with MACE allows an affordable, solution-based synthesis of SiNW arrays.^18,19,25,26^ During colloidal lithography, typically monodisperse, spherical nano- or microparticles are self-assembled and deposited onto a solid substrate^32^ followed by shrinking of the particles using oxygen plasma to obtain a non-close packed arrangement.^33^ After the template preparation, a noble metal film is deposited on top of the substrate by sputtering or thermal evaporation. The non-close packed colloidal particles act as a shadow mask and a metal film perforated with nanoholes is produced.^34^ Such metal nanohole arrays on silicon can be utilized as catalysts for MACE, where the substrate is immersed into a solution containing hydrogen fluoride (HF) and hydrogen peroxide (H_2_O_2_).^25,26^ During MACE, the silicon in contact with the noble metal film is dissolved and the metal nanohole array sinks into the surface. No etching occurs in the areas uncovered by the gold film, *i.e.* the nanoholes. As a result, nanowire arrays are formed into the silicon substrate where the length of the SiNW is controlled by the MACE duration time.^18,19,25,26^

The geometrical parameters of the resulting SiNW are predetermined by the colloidal mask. To etch SiNW by MACE, a non-close packed array of particles is required, which is frequently obtained by shrinking a hexagonal monolayer of spherical polymeric particles by oxygen plasma. The center-to-center distance between the resulting SiNW can be adjusted by the particle size, while their diameter and spacing is controlled by the plasma etching duration.^33^ Importantly, the initial diameter determines the spacing of the non-close packed particles and thus the lattice constant and diameter of the SiNW cannot be changed independently.^35^ Furthermore, the templating particles change their shape during plasma etching from a sphere to an oblate and may disintegrate when shrunken by more than half their diameter.^33^ Noteworthily, the etching process is susceptible to defects in the mask such as grain boundaries as they may lead to a breakage and delamination of the noble metal film catalyst.^36^ Therefore, high-quality monolayers are required for a reliable fabrication process.^37^ Alternatively, non-close packed masks can be directly obtained by depositing charged particles from fluid–fluid interfaces,^17,38^ or by self-assembling core–shell particles assembled at liquid interfaces.^19,39^ The assembly of core–shell particles not only allows individual control over their diameter and spacing by controlled compression at the air/water interface using a Langmuir trough,^19,39^ but also improves the quality of the interfacial monolayer as lattice defects are suppressed by the soft nature of the core–shell particles.^36,39^ Additionally, complex structural motives beyond hexagonal symmetries are possible either by tuning the particle interaction potential^40–43^ or by sequential depositions,^44^ which can be important to alter the resulting optical properties of the resulting nanowire arrays.^2,44^

While these developments increased the flexibility of colloidal lithography in combination with MACE, it is still very challenging to vary the cross-section and cylindrical shape of the SiNW, inherited by the spherical colloidal template. SiNWs with hexagonal cross-sections have been produced from colloidal monolayers by using isotropic oxygen plasma etching, which is slower at the contact points between the colloidal particles.^45^ Unfortunately, the hexagonal faceting occurs only in a narrow size window during colloidal particle shrinking and is coupled to the spacing of the colloidal particles, which limits the versatility of this approach.^45^ Silicon pillars with elliptical cross-sections have been produced using reactive ion etching and a flexible PDMS stamp,^46^ which was fabricated *via* colloidal lithography, stretched and imprinted into a polystyrene (PS) film on silicon.^47^ However, the etching mask diminishes during RIE and results in the formation of cones after extended etching time.^46^ Longer elliptical SiNWs, however, may show interesting functional properties, such as anisotropic surface reflection,^46^ polarization-dependent optical responses suitable for polarization-resolved imaging^48^ and anisotropic bundling due to capillary forces.^49^

Here we report a simple, large-scale approach to produce SiNW arrays with tunable anisotropic cross-sections using colloidal lithography and MACE. We start by the deposition of silica–poly(*N*-isopropylacrylamide) (PNIPAM) core–shell particles onto a Si wafer followed by the removal of the organic shell using oxygen plasma, resulting in a hexagonal non-close packed array of silica spheres. Next, we evaporate a thin Au layer under an oblique angle, which serves as catalyst during the MACE. After removing the silica particles, an Au film with an elliptical nanohole array is obtained. Using MACE, an array of SiNW with elliptical cross-section is obtained, where the aspect ratio is controlled by the evaporation angle. We further observe that such elliptical SiNWs preferably bend in the direction of their short axis and thus bundle into anisotropic oriented meso-structures upon exposure to capillary forces induced by solvent evaporation.^49–54^ Additionally, crescent-shaped nanowires with tunable thickness can be obtained by MACE etching without removing the spherical silica mask as the gold on top retains during the etching process. These results enrich the library of possible SiNW shapes that can be obtained from simple colloidal masks.

## Experimental

### Materials

All chemicals were purchased from commercial sources. *N*,*N*′-Methylenebis(acrylamide) (BIS; 99%, Sigma Aldrich), ammonium persulfate (APS, Sigma Aldrich, 98%), ethanol (EtOH, Sigma Aldrich, 99.9%), tetraethyl orthosilicate (TEOS; 98%, Sigma Aldrich), ammonium hydroxide solution (28–30% NH_3_ basis, Sigma Aldrich), (trimethoxysilyl)propyl methacrylate (MPS; 98%, Sigma Aldrich), (3-aminopropyl)triethoxysilane (APTS; Sigma Aldrich, >98%), hydrofluoric acid (HF, AnalaR NORMAPUR, VWR, 40%), hydrogen peroxide (H_2_O_2_, EMSURE, Merck, 30%), potassium iodide (puriss. p.a., reag. ISO, reag. Ph. Eur. 99.5%, Sigma Aldrich), iodine (99.8%, crystals, ACS, resublimed, VWR) were used as received. N-doped silicon wafers ((100), resistivity 1–30 Ω cm) were commercially purchased from Si Materials, Germany. *N*-Isopropylacrylamide (NIPAM; 97%, Sigma Aldrich) was purified by recrystallization from hexane (95%, Sigma Aldrich). Water was double deionized using a Milli-Q system (18.2 MΩ cm, Elga™ PURELAB™ Flex).

### Synthesis

SiO_2_–PNIPAM core–shell particles were synthesized, self-assembled at the air/water interface and used as masks as described in detail elsewhere.^39^ In short, silica nanoparticles with a diameter of 160 nm (±10 nm) were prepared according to the Stöber process. The silica nanoparticles were functionalized with MPS under stirring at room temperature for at least 1 d and subsequent boiling for 1 h to ensure successful functionalization. Afterwards, the particles were purified by centrifugation and redispersed three times in ethanol and three times in Milli-Q water. A PNIPAM microgel shell was polymerized on the synthesized silica nanoparticles *via* surfactant-free precipitation polymerization using 2.5 mol% BIS as crosslinker and APS as initiator.^39^ The core–shell particles were purified by centrifugation and redispersion with Milli-Q water.

### Self-assembly

The SiO_2_–PNIPAM core–shell particles were self-assembled at the air/water interface using a Langmuir–Blodgett trough (KSVNIMA) (area = 243 cm^2^, width = 7.5 cm) with Delrin barriers and the surface pressure was measured by a Wilhelmy plate. N-type silicon wafers were cut to 8 × 1.5 cm^2^ and cleaned by ultrasonication in ethanol and Milli-Q water, followed by oxygen plasma (Diener). The substrate was mounted in a 45° angle and the trough was filled with Milli-Q water. The core–shell particle suspension was diluted to 0.5 wt% and spread at the air/water interface using 30 wt% ethanol as spreading agent. After 10 min of equilibration, the barriers were compressed to a constant surface pressure of 7 mN m^−1^ and the substrate was lifted with a speed of 0.8 mm min^−1^.

### Gold evaporation

Oxygen plasma (Diener electronic Femto) was used to remove polymer residuals from the core–shell particles. Afterwards, the substrates were cut into ∼1.5 × 2 cm^2^ pieces. Titanium (HMW Hauner, 99.995%, <3 mm granulate) was evaporated under a 0°–65° angle with respect to the surface normal as an adhesion layer, subsequently followed by the evaporation of gold (HMW Hauner, 99.99%, <3 mm granulate) using a custom build thermal evaporator (Torr International Inc., THE3-KW). For an evaporation angle of 0°, 3 nm Ti and 20 nm Au were evaporated. For evaporation angles between 30°–65°, 5 nm Ti and 40 nm Au were evaporated. The silica spheres were removed using adhesive tape. Finally, the substrates were cleaned from residual tape with oxygen plasma using an Emitech K1050X at 50 W for 5 min with an oxygen flow rate of 10 mL min^−1^. The experimental details for each sample are shown in Table 1.

**Table 1:** Etching conditions and dimensions of the various SiNW arrays synthesized

Figure	Evaporation angle [°]	Sphere removal	MACE duration [min]	Cross section	SiNW length [μm]	Bundling	Long axis [nm]	Short [nm]	Aspect ratio [−]
3a and b	0°	Yes	3	Circular	∼1.6	No	163 ± 7	163 ± 7	1.0
3a and b	45°	Yes	5	Elliptical	∼2.8	No	247 ± 12	161 ± 9	1.6
3a and b	55°	Yes	5	Elliptical	∼3.0	No	303 ± 15	153 ± 14	2.0
3a and b	65°	Yes	3	Elliptical	∼1.5	No	327 ± 17	126 ± 10	2.6
3c	55°	Yes	5	Elliptical	∼3.0	No	303 ± 15	153 ± 14	2.0
3d	55°	Yes	3	Elliptical	∼0.4	No	311 ± 11	149 ± 8	2.1
4a and b	0°	Yes	10	Circular	∼9.0	Yes	159 ± 8	159 ± 8	1.0
4c–f	65°	Yes	15	Elliptical	∼8.0	Yes	336 ± 14	133 ± 10	2.6
5b and c	30°	No	3	Crescent	∼1.3	No	—	—	—
5e and f	55°	No	3	Crescent	∼1.0	No	—	—	—

### Metal-assisted chemical etching (MACE)

Si nanowires were obtained using MACE in a HF/H_2_O_2_ mixture. **Caution**: Appropriate safety precautions have to be observed when working with hydrofluoric acid (HF): HF is a contact poison! All HF steps were performed inside a HF specific fume hood, using HF resistant plastic or Teflon tools, beakers and butyl gloves. MACE was performed within a Teflon beaker using a lab-made 3D printed polymer (polylactic acid, PLA) sample holder perforated with small holes. The etching solution was prepared fresh and was composed of 10 mL HF (40%, 10.8 M), 10 mL of Milli-Q water, and 1 mL H_2_O_2_ (30%, 0.47 M). The sample was placed on the Teflon holder and immersed in the etching solution for 3–20 min, depending on the desired SiNW length (details in Table 1). Afterwards the sample was rinsed with deionized water three times, rinsed with ethanol once, and dried in air.^45^ At the end of the experiment, all pieces of equipment used were fully immersed into calcium chloride solution, thoroughly rinsed with deionized water, dried and stored inside a fume hood until further use. The Au layer was removed using a KI/I_2_ solution (10 wt% KI, 5 wt% I_2_) within 20–30 min. The SiNW array was characterized using an SEM (Zeiss, Gemini) using 5 kV and the in-lens detector. The tilted samples were taken under a 30° angle. The dimensions of the hole layer and the SiNWs were measured using ImageJ.

## Results and discussion

**Fig. 1:**
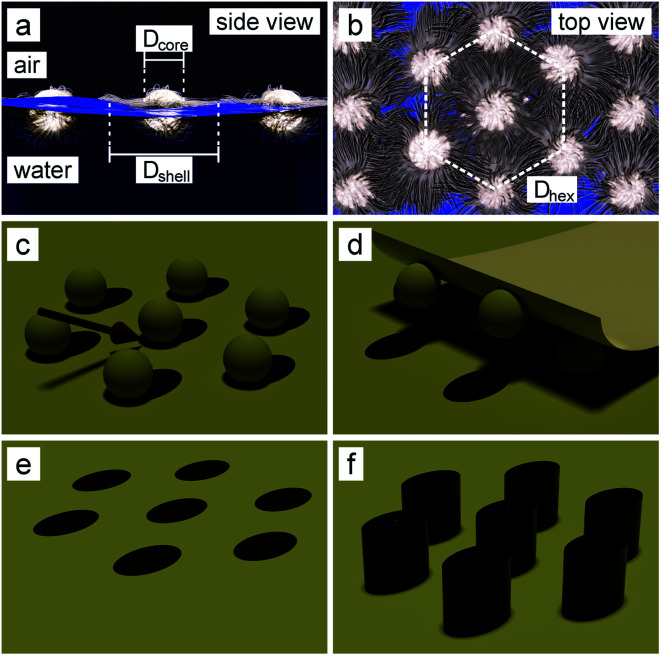
Schematic illustration of the colloidal lithography approach to obtain elliptical SiNW arrays. (a and b) SiO_2_–PNIPAM core–shell particles are self-assembled into a hexagonal lattice at the air/water interface. (c) After transfer of the colloidal monolayer onto a silicon substrate and removal of the polymeric shell by oxygen plasma, an Au layer is evaporated under an oblique angle, resulting in an elliptical shadow cast by the colloidal particle. (d and e) After removal of the colloidal particles by adhesive tape, the elliptical hole array mask is obtained. (f) Fabrication of SiNW arrays with an elliptical cross-section using metal-assisted chemical etching (MACE).

In the first step, we synthesize SiO_2_–PNIPAM core–shell particles by seeded precipitation polymerization as reported in our previous publication.^39^ The core–shell particles contain a silica core with a diameter *d*_c_ = 160 nm and a PNIPAM shell, crosslinked with 2.5 mol% *N*,*N*′-methylenebis(acrylamide). We then spread the particles at the air/water interface of a Langmuir trough, where they self-assemble into a hexagonal lattice (Fig. 1a and b). Importantly, the polymer part of the core–shell particles deform and elongate at the interface into a characteristic “fried-egg shape” or core-corona shape,^41,55,56^ where the cores are separated by the polymeric shell. The assembly is transferred onto a silicon wafer at a surface pressure of 7 mN m^−1^ to ensure a constant spacing of around 1140 nm between the SiO_2_ particles.^39^ The organic shell is subsequently removed by oxygen plasma, producing non-close packed hexagonal array of SiO_2_ particles. The advantage of the described method is the independent control of particle diameter and spacing. The SiO_2_ core diameter can be adjusted during the synthesis while their spacing can be tuned by the shell properties (thickness, crosslinking density and surface pressure during deposition).^39,41,55^ Thus, the deposited particles cannot only be precisely separated into lattices with large interparticle distances, but they also remain completely spherical in shape, which are key requirements for the next steps.

**Fig. 2:**
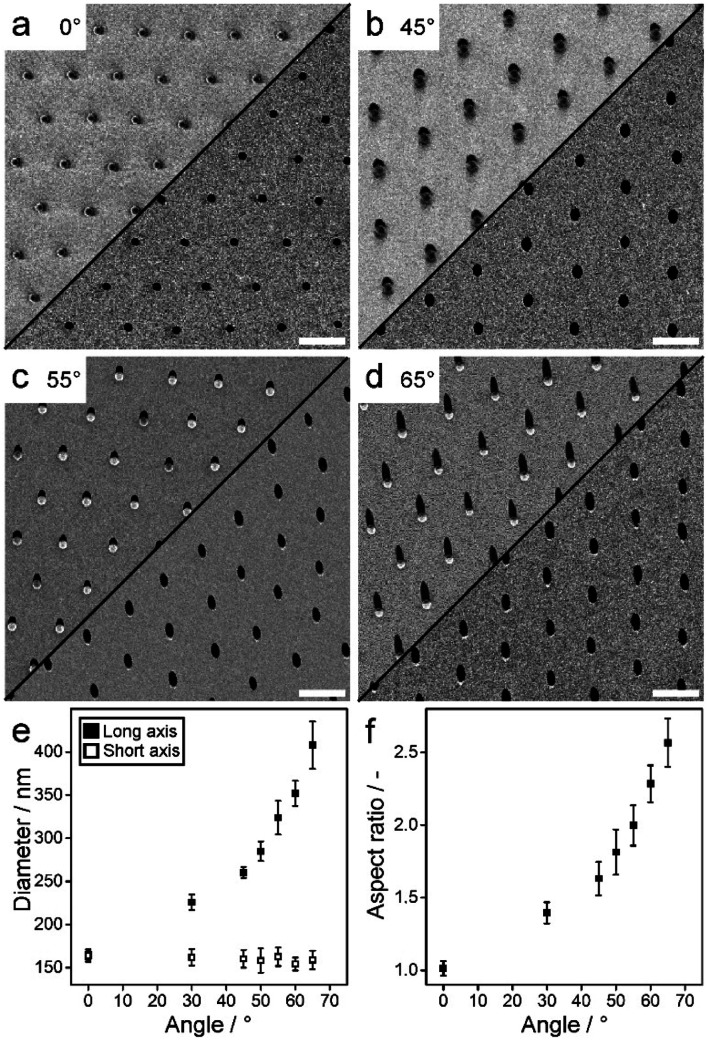
Elliptical nanohole arrays to be used as etching masks in the MACE process, formed by oblique evaporation of a Ti/Au layer onto a non-close packed colloidal monolayer. (a–d) SEM images of the metal nanohole arrays. (Top) Prior to the removal of the silica spheres and (Bottom) after removing the silica spheres using adhesive tape. Scale bars: 1 μm. (e and f) Measured short and long cross-sectional diameter and respective aspect ratio as a function of evaporation angle.

**Fig. 3:**
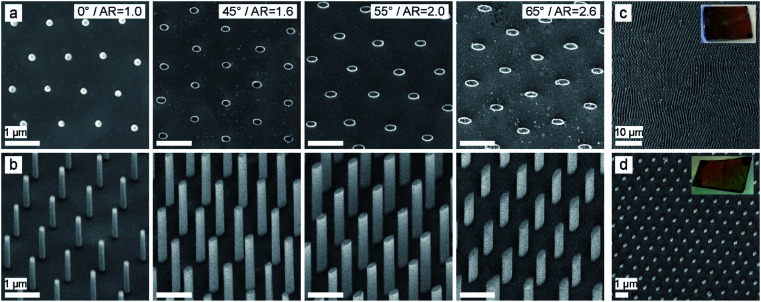
Vertically-aligned SiNW arrays with tunable, anisotropic cross-section defined by the evaporation angle of the metal film on the colloidal mask. Spherical cross-sections result from normal evaporation (0°); elliptical cross-sections with tailored aspect ratio result from oblique evaporation (45°, 55°, 65°). The aspect ratio (AR) varies from 1–2.6. (a) Top view SEM images. (b) 30° tilted SEM images. The length of the SiNW is between 2–5 μm. Scale bars: 1 μm. (c and d) Low-magnification 30° tilted SEM images and photograph of the etched Si wafer of elliptical SiNWs with an AR of 2.0, etched for 5 min (length ∼ 3 μm) (c) and for 3 min (length ∼ 0.4 μm) (d). The Au layer was removed using a KI/I_2_ solution.

In the next step, 20–40 nm Au with 3–5 nm Ti as adhesion layer is thermally evaporated under an oblique angle (Figure 1c and 2a–d: top). After removal of the SiO_2_ particles using adhesive tape (Fig. 1d), an elliptical hole mask is obtained (Figure 1e and 2a–d: bottom). We then etch elliptical SiNWs using MACE by dipping the substrates into an aqueous solution containing 10 : 1 : 10 HF : H_2_O_2_ : H_2_O for 3–20 minutes, depending on the desired SiNW length. Hereby the Au layer catalyses the dissolution of silicon beneath the layer,^25,26^ leading to the etching of ordered, vertically-aligned SiNWs (Fig. 1f and 3). The aspect ratio (AR) of the elliptical cross-section, and with this the shape of the resulting nanowire arrays (Fig. 3a and b), can be tuned by the evaporation angle up to 2.6. Additionally, low magnification SEM images and photographs of the etched substrates show the high homogeneity and reliability of the etching process (Fig. 3c and d).

After MACE, the substrates are thoroughly washed in ethanol and left to dry. The evaporation of the solvent trapped within the array can generate strong capillary forces associated with the liquid/vapor meniscus between the tips of the SiNWs. This can cause the deformation and even collapse of the SiNWs, leading to bundling.^57–59^ Alternatively, supercritical CO_2_ drying can be used to reduce the occurrence of bundling.^60^ The bundling can be described by the competition between the capillary force (*F*_C_), which attracts neighboring pillars, and the elastic deformation force (*F*_E_), which resists the bending.^50^ The capillary force depends on the surface tension of the liquid (*γ*), the height (*h*) and the long axis of the SiNW (*a*), the solid–liquid contact angle (*θ*) and the distance between the SiNW (*s*) (*F*_C_ ∼ *γha* cos(*θ*)*s*^−1^).^49^ The elastic deformation force is *F*_E_ ∼ *EI*δ*xh*^−3^, where *E* is the Young's modulus, *I* is the second moment of area and δ*x* the deformation length.^61^ Bending of two neighboring wires towards each other results when *F*_C_ > *F*_E_. The capillary-induced bundling behavior of pillars with various cross-sections has been previously investigated for polymeric pillar arrays obtained by laser printing^49,52–54^ or photolithography.^50,51^ The flexible nature of the polymeric pillars (*E* ∼ 0.1–2 GPA) allowed the pillars to not only bend but also twist into hierarchical helical assemblies.^50^ Here we investigate the capillary-induced bundling behavior of much stiffer SiNWs (*E* ∼ 130–170 GPA)^62^ as a function of cross-sectional shape.

**Fig. 4:**
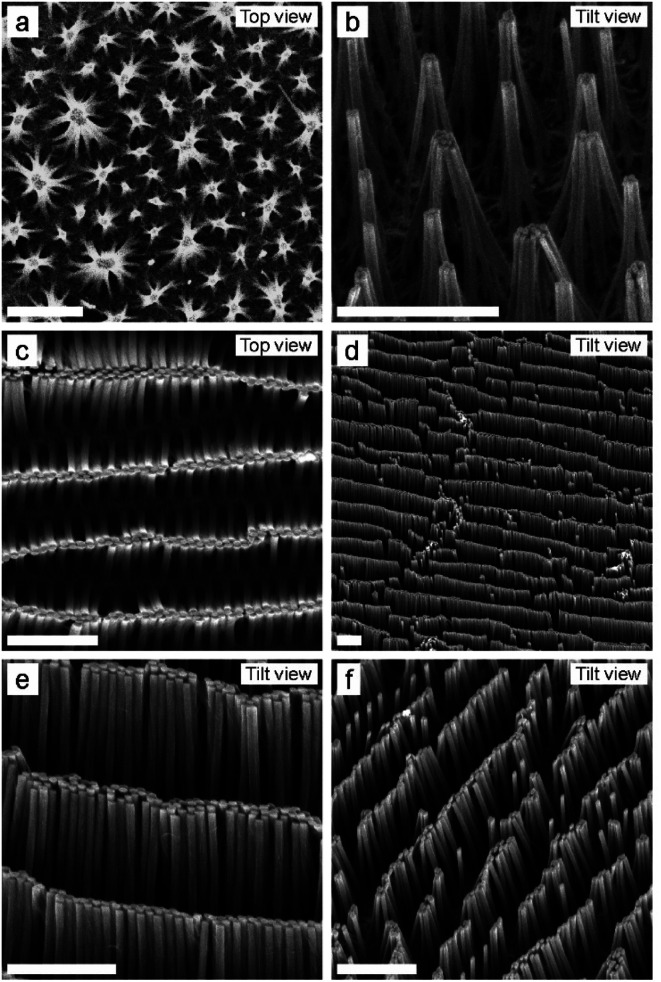
Anisotropic mesostructures created by controlling capillary-induced pillar collapse. (a and b) Nanowires with a spherical cross-section bundle with their closest neighbors to a circular, “flower-like” mesostructure. (c–f) Nanowires with an elliptical cross-section preferably bend in the direction of their minor axis as the respective bending modulus scales with the thickness of the nanowires. This leads to defined bundling into oriented, anisotropic linear mesostructures. Scale bar: 5 μm.

For a SiNW with a rotationally symmetric circular cross-section, *I* = ¼π*r*^4^, where *r* is the cylinder radius, and thus their bending direction should be random. The equal distance between the SiNWs leads to the bundling into “flower-like” structures (Fig. 4a and b). Noteworthily, the high Young's modulus of the SiNW prevents them from twisting into hierarchical helical assemblies. On the other hand, for SiNWs with an elliptical cross-section, *F*_E_ and *F*_C_ depend on the bending direction. The second moment of area of the SiNW with an elliptical cross-section bending into the direction of the long axis is *I*_a_ = ¼π*a*^3^*b*, and in the direction of the short axis *I*_b_ = ¼π*ab*^3^, where *a* is the long axis and *b* the short axis. Thus, the elastic force is smaller in the direction of the short axis and the pillars preferably bend in this direction.^49^ This preferential bending direction leads to anisotropic, linear hierarchical mesostructures, which are oriented along the direction of the long axis of the SiNW (Fig. 4c–f). Such oriented SiNW mesostructures may have potential application in nanobiotechnology, as they may guide the growth of cells.^63,64^ Furthermore, their anisotropic topography may produce anisotropic wettability and guide the flow of water droplets, comparable to the natural example of the rice leaf, which has a similar morphology.^65–67^

**Fig. 5:**
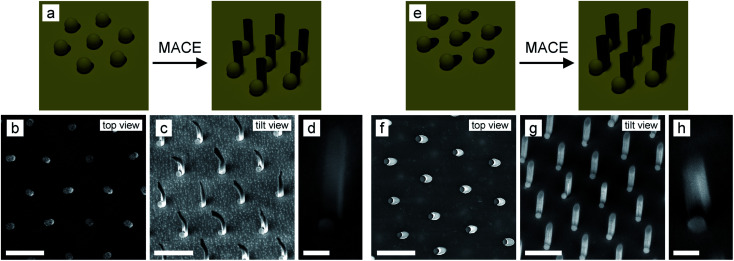
Process to obtain SiNW with a crescent-shaped cross-section with a thin wall thickness (a–d) and thick wall thickness (e–h). (a and e) Schematic illustration of the etching process to obtain crescent-shaped SiNW with different wall thicknesses. (b–d) SEM images of thin crescent-shaped SiNW evaporated under a 30° angle. (f–h) SEM images of thick crescent-shaped SiNW evaporated under a 55° angle. The Au layer was removed using KI/I_2_ solution. (c, d, f and h) Scale bar: 1 μm. (d and h) Scale bar: 200 nm.

Last, we demonstrate the fabrication of SiNWs with a crescent-shaped cross-section (Fig. 5). To this end, we follow a similar etching protocol but do not remove the SiO_2_ particles after angular evaporation of the Au layer. Instead, we use the patterns as shown in Fig. 2 (top images) and perform the MACE process as schematically illustrated in Fig. 5a and e. When dipped into the MACE solution, the SiO_2_ particles are immediately dissolved by HF, but the Au cap, which covered the sphere, remains attached to the Au layer. During the sinking of the Au layer, the Au caps come into contact with the elliptical SiNW and etch a circular hole into the elliptical SiNW. Therefore, we obtain crescent-shaped SiNWs, where the wall thickness can be adjusted by the evaporation angle (Fig. 5). While the SiNWs evaporated under a 55° angle remain stable, the thinner SiNWs evaporated under a 30° angle bend preferentially in the concave direction of the crescent. Interestingly, since the Au catalyst layer is now not completely flat but has a 3D structure (the film is increased by 160 nm at the position of the SiO_2_ particles), the resulting SiNW array is also not fully flat at the bottom. After removal of the Au layer by a KI/I_2_ solution, there is still a spherical attachment at the bottom of each SiNW, reminiscent of the elevated topography of the initial colloidal particle (Fig. 5b–d and f–h).

## Conclusion

To summarize, we developed a simple and affordable technique to synthesize large-scale, homogeneous SiNW arrays with tunable anisotropic cross-sections. The approach utilizes the shadow of the templating colloidal particles under angular thermal evaporation, where the aspect-ratio of the elliptical holes can be adjusted with the evaporation angle. When the templating spheres are removed, the metal-assisted chemical etching results in SiNWs with elliptical cross-sections or, when the templating spheres remain on the surface, in nanowires with crescent shaped cross-sections. We further demonstrate the anisotropic bundling of nanowires with elliptical cross-sections upon capillary-induced collapse. The anisotropic collapse along their short axis results in anisotropic, linear mesostructures. These results demonstrate that a rich library of SiNW shapes and mesostructures is possible using simple spherical colloidal particles as masks.

## Funding sources

The research was supported by the Deutsche Forschungsgemeinschaft (DFG) under grant number VO 1824/6-2. M. R. acknowledges funding from the Swiss National Science Foundation Project-ID P2SKP2_194953 and temporary funding from EAM. N. V. also acknowledges support by the Interdisciplinary Center for Functional Particle Systems (FPS). G. R. B. and J. F. W. gratefully acknowledge support from the Austrian Science Fund FWF for project P-33159.

## Author contributions

J. J. T. synthesized the core–shell particles. R. S. B and M. R. self-assembled and deposited the particles onto a substrate. E. S. A. G. evaporated the catalyst layers. F. J. W. and M. R. etched the SiNW using MACE. M. R., F. J. W., G. B. and N. V. designed the experiments and the study. All authors contributed to the writing of the manuscript.

## Conflicts of interest

There are no conflicts to declare.
